# Growth of infants fed formula supplemented with *Bifidobacterium lactis* Bb12 or *Lactobacillus* GG: a systematic review of randomized controlled trials

**DOI:** 10.1186/1471-2431-13-185

**Published:** 2013-11-12

**Authors:** Hania Szajewska, Anna Chmielewska

**Affiliations:** 1 Department of Paediatrics, The Medical University of Warsaw, Dzialdowska 1, Warsaw 01-183, Poland

**Keywords:** Feeding, Growth, Probiotics, Infants, Children

## Abstract

**Background:**

Growth is an essential outcome measure for evaluating the safety of any new ingredients, including probiotics, added to infant formulae. The aim of this systematic review was to determine the effects of supplementation of infant formulae with *Bifidobacterium lactis* Bb12 *(B lactis)* and/or *Lactobacillus rhamnosus* GG (LGG) compared with unsupplemented formula on the growth of healthy infants.

**Methods:**

The MEDLINE, EMBASE, and Cochrane Library databases were searched in June 2013 for relevant randomized controlled trials (RCTs) conducted in healthy term infants. Unpublished data were obtained from the manufacturer of *B lactis*-supplemented formula. The primary outcome measures were weight, length, and head circumference.

**Results:**

Nine eligible trials were identified. Compared with unsupplemented controls, supplementation of infant formula with *B lactis* had no effect on weight gain [4 RCTs, n = 266, mean difference (MD) 0.96 g/day, 95% confidence interval (CI) -0.70 to 2.63)], length gain (4 RCTs, n = 261, MD −0.39 mm/month, 95% CI −1.32 to 0.53), or head circumference gain (3 RCTs, n = 207, MD 0.56 mm/month, 95% CI −0.17 to 1.30). Data limited to one small (n = 105) trial suggest that infants who received standard infant formula supplemented with LGG grew significantly better. No such effect was observed in infants fed hydrolyzed formula supplemented with LGG.

**Conclusions:**

Supplementation of infant formula with *B lactis* results in growth similar to what is found in infants fed unsupplemented formula. Limited data do not allow one to reach a conclusion regarding the effect of LGG supplementation on infant growth.

## Background

Growth is a sensitive, although nonspecific, sign of the overall health and nutritional status of an infant. It is also an essential outcome measure for evaluating the safety of any new ingredient added to infant formulae such as probiotics. Generally, growth studies should include at least measurements of weight and length velocity and head circumference [1].

In 2010, the Committee of Nutrition of the European Society for Paediatric Gastroenterology, Hepatology and Nutrition (ESPGHAN) commented on infant formulae supplemented with probiotics (and/or prebiotics) [2]. Based on the evidence searched up to January 2010, it was concluded that these formulae do not raise safety concerns with regard to growth. The Committee evaluated only studies in which infant formulae were supplemented with probiotics and/or prebiotics during the manufacturing process. Studies in which probiotics/prebiotics were not introduced during the manufacturing process, but administered thereafter, for example in capsules, the contents of which were added to infant formulae, were excluded. Given this, and considering the fact that new studies have been published, the present review was undertaken to update data on the efficacy of using probiotic-supplemented formulae. The main objective was to determine the effects of supplementation of infant formulae with *Bifidobacterium lactis* Bb12 *(B lactis)* and/or *Lactobacillus rhamnosus* GG (LGG) compared with unsupplemented formula administered in early infancy (starting <4 months of age) on the growth of healthy infants. The choice of the probiotic strains was determined by the facts that both are widely available in many countries and are commonly used in (or with) infant formulae.

## Methods

For this systematic review, we followed the methods described elsewhere [2]. In brief, the Cochrane Central Register of Controlled Trials (CENTRAL, the Cochrane Library), MEDLINE, and EMBASE databases were systematically searched in June 2013 by both authors independently, with no language restrictions. The following search terms were used in different combinations: *Bifidobacterium* or *Bifidobacterium bifidum* or *B bifidum* or *B. bifidum* or *B lactis* or *B. lactis* or *B lactis* Bb1*2* or *B. lactis* Bb12 or *Bifidobacterium animalis* or *B. animalis* or *Bifidobacterium animalis* ssp lactis or CNCMI-3446 or *Lactobacillus* or *Lactobacillus rhamnosus* or *Lactobacillus rhamnosus* GG or *L. rhamnosus* GG or LGG; formula or formulae or milk; growth or anthropometry or weight or length or head circumference or development or physical development; newborn or infant or infant* or infants or child or children or child*. For the full PubMed electronic search strategy, see Additional file 1: Table S1. The reference lists from identified studies and key review articles were also searched. Letters to the editor and abstracts from scientific meetings were excluded unless a full set of data was obtained from the authors. Nestlé Nutrition Institute (NNI) was contacted for unpublished data.

The search was restricted to randomized controlled trials (RCTs) carried out in healthy term infants. Participants in the experimental group received infant formulae supplemented with *B lactis* (depending on taxonomic classification also known as *B bifidum, B lactis* Bb12, *B animalis* ssp *lactis* CNCMI-3446), *Lactobacillus rhamnosus* GG ATCC 53103 (LGG), or a combination of these 2 strains. Studies in which probiotics were either added to a formula during the manufacturing process or were administered separately, for example in capsules, the contents of which were added to infant formula, were considered for inclusion. Formulae manufactured from cow’s milk proteins or any other proteins, as well as formulae based on protein hydrolysates, were eligible for inclusion. Subjects in the experimental group received the study formula with probiotic(s), and subjects in the control group received the same formula without probiotic supplementation. If other comparisons were made (for example, one trial [3] used formula mixed with another probiotic strain [*L reuteri* ATCC 55730] as a control), these other arms are not evaluated here. This is because the objective of this review was to evaluate the effect, if any, of supplementation of infant formula with probiotics such as *B. lactis* or LGG or their combinations only, and not of other formula differences. Also, we did not evaluate here breast-fed reference groups. The administration of infant formula had to start in early infancy (below 4 months of age).

The primary outcome measures of interest were growth parameters recommended by the Institute of Medicine (IOM), i.e., weight, length, and head circumference. The secondary outcome measures were body mass index (BMI), body composition, skinfold, and dual-energy X-ray absorptiometry (DXA) [1].

Titles and abstracts of all identified studies were screened, and the full text of each potentially relevant trial was retrieved. The reviewers independently applied the inclusion criteria to each trial assessed as relevant. Differences in opinion concerning the eligibility of the studies for the review were resolved by discussion. Data extraction was performed with use of standard data-extraction forms. We contacted by email the authors of the studies that reported growth but did not provide data. However, we failed to obtain additional growth data. NNI provided us with data from one clinical trial that was published as an abstract only [4]. Moreover, in the study by *Urban* et al. [5], unlikely data of head circumference increments of about 4.7 cm per month were found. These data were compared with the original statistical report made available to us by NNI. As it turns out, by mistake, instead of showing data on head circumference, the authors showed data on BMI. Here, the correct data are presented.

The risk of bias in the studies meeting the inclusion criteria was assessed independently by the reviewers with the implementation of The Cochrane Collaboration’s tool for assessing risk of bias. The following criteria were used: adequacy of sequence generation, allocation concealment, and blinding of participants, personnel and outcome assessors; and extent of loss to follow-up, i.e., the proportion of patients in whom the investigators were not able to determine outcomes (incomplete outcome data). Low risk of bias was indicated by an answer of ‘*yes*’, and a high risk, by an answer of ‘*no*’ [6].

The data were analyzed using RevMan ([Computer program]. Version 5.2. Copenhagen: The Nordic Cochrane Centre, The Cochrane Collaboration, 2012). The mean difference (MD) between the treatment and control groups was selected to represent the difference in continuous outcomes (with 95% confidence interval, CI). Heterogeneity was quantified by *χ*^2^ and *I*^2^, which can be interpreted as the percentage of the total variation between studies that is attributable to heterogeneity rather than to chance. A value of 0% indicates no observed heterogeneity, and larger values show increasing heterogeneity. If heterogeneity was not revealed, we present results of only the fixed effects model. If there was substantial heterogeneity (over 50%), the analyses were based on the random effects model. Although funnel plots to determine publication bias were planned, there were too few studies to warrant generation of a funnel plot.

## Results

For a flow diagram documenting the identification process for eligible trials, see Additional file 2: Figure S1. Table 1 summarizes the key characteristics of included trials. Among them, 7 RCTs assessed the effects on growth of infant formulae supplemented with *B lactis* during early infancy [3-5,7-10]. These formulae differed mainly with regard to protein (although all had similar energy densities through adjustment of the fat content), and included infant formula with 2.2 g protein/100 kcal [3]; reduced protein (1.8 g protein/100 kcal) infant formula [4]; reduced protein (1.8 g protein/100 kcal) infant formula supplemented with long-chain polyunsaturated fatty acids [7]; acidified infant formula (2 g protein/100 kcal) [5,9]; partially hydrolyzed 100% whey formula (2.2 g protein/100 kcal) [8]; and reduced protein (1.9 g protein/100 kcal), partially hydrolyzed 100% whey formula [10]. Two RCTs evaluated the effects on growth of formulae supplemented with LGG (standard infant formula [11], extensively hydrolyzed casein formula, and partially hydrolyzed whey-casein [60:40] formula [12]. Two RCTs that evaluated the effects of the administration of a combination of LGG and *B lactis* were identified, but growth was not assessed [13,14]. An attempt was made to contact the authors, but with no success. Thus, these 2 RCTs were excluded. See Additional file 3: Table S2 for a complete list of excluded trials with reasons for exclusion.

**Table 1:** Characteristics of included trials

**Reference (country)**	**Participants (age at enrollment)**	**Intervention**	**Comparison**	**Duration of intervention (follow-up)**
***B LACTIS***				
Barclay 2003 (Italy) [4] plus unpublished data	Healthy term newborns, ≤ 28 d of life at enrollment;	Bb12 (3 × 10^7^ CFU) in reduced protein (1.8 g/100 kcal) IF (n = 29/53)	Reduced protein (1.8 g/100 kcal) IF (n = 27/58)	4 mo (4 mo)
	BW 2500 to 4200 g			
Gibson 2009 (Australia) [7]	≥37 wk gestation, BW 2500–4500 g, ≤10 d	Bb12 (3.85 × 10^8^ CFU/100 kcal + LCPUFA in reduced protein IF (1.8 g protein/100 kcal) (n = 62/72)	Standard IF (n = 62/70)	7 mo (7 mo)
Holscher 2012 (USA) [8]	Healthy term infants (7 wk)	Bb12 (10 ^6^ CFU/g) in pHF 100% whey (2.2 g protein/100 kcal) (n = 41/50)	pHF 100% whey (n = 34/43)	6 wk (6 wk)
Urban 2008 (South Africa) [5] plus unpublished data	Healthy term infants born to HIV-infected mothers (≤ 1 wk)	Bb12 (? CFU-no data*)* in acidified IF (2 g protein/100 kcal) (n = 29/45*)	Acidified IF (n = 28/43*)	119 d (182 d)
Velaphi 2008 (South Africa) [9]	Healthy term infants born from HIV(+) mothers (≤1 wk)	Bb12** (? CFU-no data) in chemically acidified IF (2 g protein/100 kcal) (n = 31/53*)	Chemically acidified IF (n = 34/51*)	6 mo (182 d)
Weizman 2006 (Israel) [3]	Healthy term infants <4 mo	Bb12 (1 × 10^7^ CFU/g) in IF (2.2 g protein/100 kcal) (n = 20)	Standard IF (n = 19)	4 wk (4 wk)
Ziegler 2003 (Germany) [10]	Healthy term infants (6–10 d)	Bb12 (3.6 × 10^9^ CFU /g) in reduced protein (1.9 g/100 kcal) 100% whey pHF (n = 28/40)	Reduced protein, 100% whey pHF (n = 27/40)	4 mo (112 d)
***LACTOBACILLUS *** **GG**				
Scalabrin 2009 (USA) [12]	Healthy term infants (14 d)	LGG (10^8^ CFU/g) in EH casein formula (n = 63/94)	EH casein formula (n = 70/94)	120 d/150 d in a subgroup
Vendt 2006 (Estonia & Finland) [11]	Healthy term infants (≤2 mo)	LGG (10^7^ CFU/g) in IF (n = 51/60)	IF (n = 54/60)	6 mo (6 mo)

Bb12, *B lactis* Bb12; BF, breastfeeding; BW, birth weight; CF, control formula; CFU, colony forming units; EH, extensively hydrolyzed; IF, infant formula; LCPUFA, long chain polyunsaturated fatty acids; LGG, *Lactobacillus rhamnosus* GG; pHF, partially hydrolyzed.

* HIV (-) with full follow-up.

** name used in the publication B lactis CNCM I 3446.

The duration of the intervention and time of follow-up ranged from 4 weeks to 7 months. The doses of the probiotic used ranged from 10^6^ to 3.6 × 10^9^ colony-forming units (CFU) per 1 gram of formula. The participants in all of the included trials were healthy infants born at term. Two studies included newborns born to HIV-positive mothers and analyzed only those infants who were HIV-negative [5,9].

Methodological quality (Table 2) varied among the studies, but in general it was moderate. In four trials [4,5,9,11], the dropout rate was very high. For example, in the study by *Urban* et al. [5], out of 88 randomized infants, only 57 (64.7%) were available for analysis; in the study by *Barclay et al.*[4], out of 111 randomized infants, only 56 (50%) were available for analysis.

**Table 2:** Methodological quality of included trials

**Reference**	**Adequate sequence generation?**	**Allocation concealment?**	**Blinding?**	**Incomplete outcome data addressed?**
***B LACTIS***				
Barclay 2003 [4] plus unpublished data	Unclear*	Yes	Yes	No**
Gibson 2009 [7]	Yes	Yes	Yes	Yes
Holscher 2012 [8]	Unclear	Unclear	Yes	Yes
Urban 2008 [5] plus unpublished data	Yes	Yes	Yes	No**
Velaphi 2008 [9]	Unclear	Unclear	Yes	No**
Weizman 2006 [3]	Yes	Unclear	Yes	Yes
Ziegler 2003 [10]	Unclear	Unclear	Unclear	No**
***LACTOBACILLUS GG***				
Scalabrin 2009 [12]	Yes	Yes	Yes	No
Vendt 2006 [11]	Yes	Unclear	Yes	Yes

In all cases, an answer of ‘yes’ indicates a low risk of bias, and an answer of ‘no’ indicates a high risk of bias.

* The randomization procedure was changed during the study because of a problem with formula supply.

** A drop-out rate >20%.

**Figure 1:**
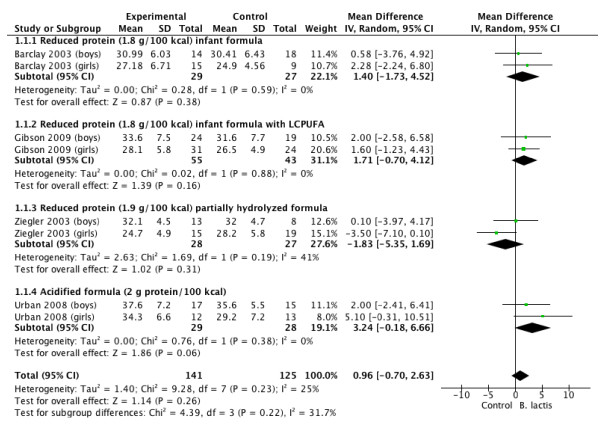
***B lactis *vs. control.** Administration started in infants <4 mo of age. Outcome: weight gain (g/day).

**Figure 2:**
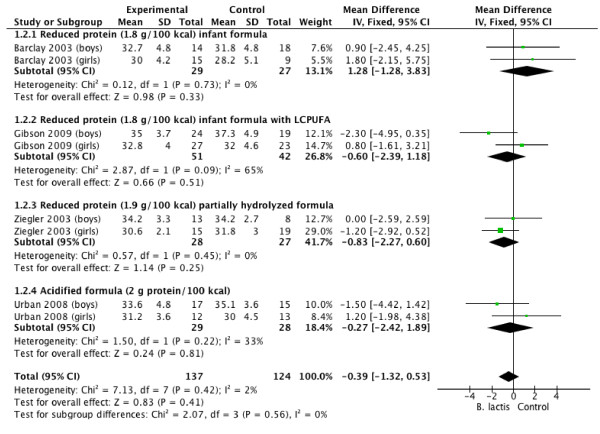
***B lactis *vs. control.** Administration started in infants <4 mo of age. Outcome: length (mm/month).

**Figure 3:**
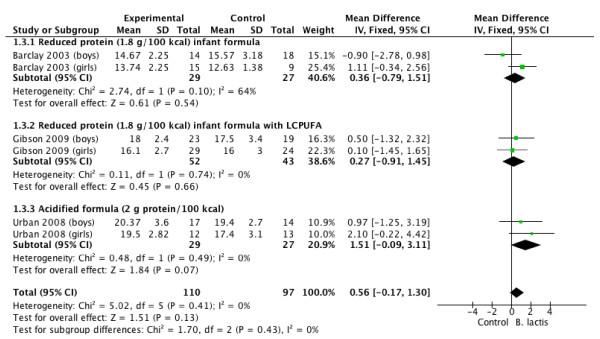
***B lactis *vs. control.** Administration started in infants <4 mo of age. Outcome: head circumference (mm/month)

**Figure 4:**
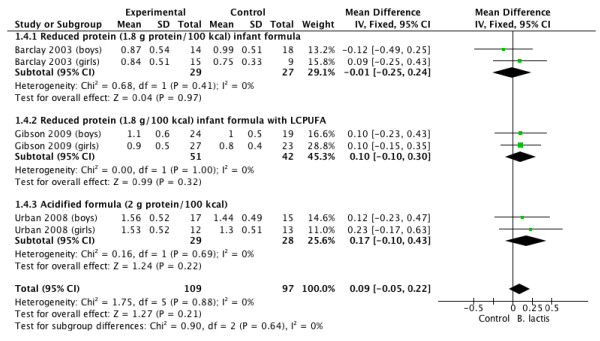
***B lactis *vs. control.** Administration started in infants <4 mo of age. Outcome: BMI (kg/m2/month).

Tables 3 and 4, and Figures 1, 2, 3 and 4 summarize the main results of this review.

**Table 3:** **Formulae supplemented with *B lactis***

**Outcome**	**RCTs**	**Participants**	**Effect estimate**
			**MD (95% CI), fixed effect model**
**Weight gain (g/day)**	4	266	0.96 (−0.70 to 2.63) (random)
• Reduced protein (1.8 g/100 kcal) IF	1	56	1.40 (−1.73 to 4.52) (random)
• Reduced protein (1.8 g/100 kcal) IF with LCPUFA	1	98	1.71 (−0.7 to 4.12) (random)
• Reduced protein (1.9 g/100 kcal) pHF	1	55	−1.83 (−5.35 to 1.69) (random)
• Acidified formula (2 g protein/100 kcal)	1	57	3.24 (−0.18 to 6.66) (random)
**Length (mm/month)**	4	261	−0.39 (−1.32 to 0.53)
• Reduced protein (1.8 g/100 kcal) IF	1	56	1.28 (−1.28 to 3.83)
• Reduced protein (1.8 g/100 kcal) IF with LCPUFA	1	93	−0.60 (−2.39 to 1.18)
• Reduced protein (1.9 g/100 kcal) pHF	1	55	−0.83 (−2.27 to 0.60)
• Acidified IF (2 g protein/100 kcal)	1	57	−0.27 (−2.42 to 1.89)
**Head circumference (mm/month)**	3	207	0.56 (−0.17 to 1.30)
• Reduced protein (1.8 g/100 kcal) IF	1	56	0.36 (−0.79 to 1.51)
• Reduced protein (1.8 g/100 kcal) IF with LCPUFA	1	95	0.27 (−0.91 to 1.45)
• Acidified IF (2 g protein/100 kcal)	1	56	1.51 (−0.09 to 3.11)
**Body mass index (BMI)(kg/m**^**2**^**/month)**	3	206	0.09 (−0.05 to 0.22)
• Reduced protein (1.8 g/100 kcal) IF	1	56	−0.01 (−0.25 to 0.24)
• Reduced protein (1.8 g/100 kcal) IF with LCPUFA	1	93	0.10 (−0.10 to 0.30)
• Acidified IF (2 g protein/100 kcal)	1	57	0.17 (−0.10 to 0.43)
**Final weight percentiles** (IF 2.2 g protein/100 kcal)	1	39	−2.7 (−13.37 to 7.97)
**Final length percentiles** (IF 2.2 g protein/100 kcal)	1	39	−2.00 (−12.75 to 8.75)
**Final HC percentiles** (IF 2.2 g protein/100 kcal)	1	39	−7.5 (−18.15 to 3.15)

HC, head circumference; IF, infant formula; LCPUFA, long-chain polyunsaturated fatty acids; MD, mean difference; pHF, partially hydrolyzed formula; RCTs, randomized controlled trials.

Summary of the results.

**Table 4:** **Formulae supplemented with *Lactobacillus *** **GG**

**Outcome**	**RCTs**	**Participants**	**Effect estimate**
			**MD (95% CI), fixed effect model**
**Weight (g)**			
• At entry	1	105	−440 (−487 to −393)
• After 3 mo	1	105	−323 (−374 to −271)
• At 6 mo	1	105	−150 (−204 to −96)
**Length (mm)**			
• At entry	1	105	−18 (−20 to −16)
• After 3 mo	1	105	−12 (−13 to −11)
• At 6 mo	1	105	−7 (−8 to −6)
**Head circumference (mm)**			
• At entry	1	105	−5 (−6 to −4)
• After 3 mo	1	105	−3 (−4 to −2)
• At 6 mo	1	105	0.0 (−0.8 to 0.8)
**Change in standard deviation score**			
• Weight after 3 mo	1	105	0.33 (0.24 to 0.42)
• Weight at 6 mo	1	105	0.44 (0.39 to 0.49)
• Length after 3 mo	1	105	0.27 (0.16 to 0.38)
• Length at 6 mo	1	105	0.37 (0.27 to 0.47)
• HC after 3 mo	1	105	0.19 (0.14 to 0.24)
• HC at 6 mo	1	105	0.27 (0.23 to 0.31)

HC, head circumference; MD, mean difference; RCTs, randomized controlled trials.

Summary of the results.

### *B lactis*

The effect of supplementation of infant formulae with *B lactis* on growth parameters was studied in 7 RCTs [3-5,7-10]; thus, in comparison with the 2010 analyses, 4 new RCTs were added to this analysis or were updated.

### Weight gain

The effect of *B lactis* supplementation on weight gain was studied in 4 RCTs [4,5,7,10]. No significant differences between the experimental groups and the control groups were reported in any of the studies. The pooled results of 4 RCTs (n = 266) revealed no significant difference in weight gain between the probiotic and control groups (MD 0.90 g/day; 95% CI −0.51 to 2.32, fixed effect model). Heterogeneity was found (*I*^*2*^ 54.1%); however, there was still no significant difference in weight gain between the probiotic and control groups in the random effect model (MD 0.96 g/d; 95% CI −0.70 to 2.63; *I*^*2*^ 31.7%) (Figure 1).

### Length gain

The effect of *B lactis* supplementation on length gain was studied in 4 RCTs [4,5,7,11]. None of the individual trials reported significant differences between the study groups. The pooled results of 4 RCTs (n = 261) also revealed that infants fed formula supplemented with *B lactis* had similar length gain compared with infants fed unsupplemented formula (MD −0.39 mm/month; 95% CI −1.32 to 0.53). No significant heterogeneity was found (*I*^*2*^ = 0%) (Figure 2).

### Head circumference gain

The effect of *B lactis* supplementation on head circumference gain was studied in 3 RCTs [4,5,7]. No significant differences between the experimental groups and the control groups were reported in any of the studies. The pooled results of the 3 trials (n = 207) revealed no significant difference between the probiotic and control groups in head circumference gain (MD 0.56 mm/month; 95% CI −0.17 to 1.30; *I*^*2*^ = 0%) (Figure 3).

### BMI

The effect of *B lactis* supplementation on BMI was studied in 3 RCTs [4,5,7]. No significant differences between the experimental groups and the control groups were reported in any of the studies. The pooled results of 3 trials (n = 206) revealed no significant difference between the probiotic and control groups in BMI (MD 0.09 kg/m^2^/month; 95% CI −0.05 to 0.22; *I*^*2*^ = 0%) (Figure 4).

### Body composition, skinfold, DXA

None of the studies reported on any of these predefined outcomes.

### Other presentations of growth outcomes

*Urban* et al. [5] reported no differences between the study groups in *Z*-scores for weight-for-age, length-for-age, and head circumference-for-age (data not shown but available upon request). *Velaphi et al.*[9] demonstrated that there was an increase in *z* scores for all studied formulae; however, no significant differences were found for weight-for-age (P = 0.22), length-for-age (P = 0.56), head circumference-for-age (P = 0.66), and weight-for-length (P = 0.13) between the study groups. In the original publication, data were presented as figures only, so data are not reported here. *Weizman et al*. [3] reported that the use of standard infant formula supplemented with *B lactis*, compared with unsupplemented standard infant formula, had no effect on growth assessed by final weight, length, and head circumference percentiles (see Additional file 4: Figure S2). *Holscher et al.*[8] reported that mean weight percentiles generated from the World Health Organization growth charts did not differ between infants fed formula with 2.2 g protein (100% partially hydrolyzed whey)/100 kcal or the same formula supplemented with *B lactis* (data not presented in the original study).

### *Lactobacillus* GG

One RCT [11] involving 105 infants fed standard infant formula supplemented with *LGG* provided data on growth. At entry, despite randomization, the groups were not equivalent. Compared with the control group, children randomly assigned to the LGG group were significantly smaller with regard to weight, length, and head circumference. These differences between groups remained significant at 3 and 6 months of age, except for head circumference at 6 months (see Additional file 5: Figure S3). However, compared with children receiving unsupplemented formulae, those receiving formula supplemented with LGG grew better, as documented by significantly higher changes in standard deviation scores (see Additional file 6: Figure S4). One further RCT investigated the impact on growth in infants fed extensively hydrolyzed casein formula with or without LGG supplementation [12]. In principle, no significant differences between the study groups were found with regard to growth rates from day 14 to day 30, 120, or 150. Data are not presented, but available in the original publication and upon request.

## Discussion

### Summary of findings

The objective of this review was to update evidence on the effects of supplementation compared with non-supplementation of infant formulae with *B lactis* and/or LGG on the growth of healthy infants. It was not designed to evaluate the effects of supplementation compared to breast milk. As it is desirable that growth measurements should be taken during the period when infant formula remains an exclusive source of nutrition for an infant, we focused on supplementation during early infancy. In general, *B lactis* supplementation results in growth similar to what is found in unsupplemented infants. Caution is needed not to over-interpret these results as in some of the studies only a subset of infants was available for analysis. With regard to LGG supplementation, data limited to only one trial suggest that infants who received infant formula supplemented with LGG grew better. The interpretation of these findings is difficult. First, the groups were not equivalent at entry into the study. Second, the mechanisms as to how LGG supplementation might influence weight and length gain are not clear. Finally, no such effect was observed in infants fed hydrolyzed formula supplemented with LGG.

None of the studies evaluated the effect of probiotic supplementation on body composition. The major advantage of this assessment is that it allows more precise assessment of the metabolic effects of ingredients. It is also considered as a potential long-term predictor of health outcomes. Body composition is, however, difficult and expensive to measure; the best method of measurement requires DXA [1].

### Strengths and limitations

This systematic review largely focuses on a single, well-defined probiotic (*B lactis*). Furthermore, it is based on the largest number of studies, and it includes unpublished data. However, there are limitations to this review. The number of trials with a particular type of probiotic and/or a specific type of formula was small. The methodological quality and the quality of reporting the study findings were variable and sometimes poor, especially in earlier published studies. Other potential limitations include unclear sequence generation, unclear allocation concealment, and a very high dropout rate in some of the included trials. The findings are, therefore, likely to be affected by a varying degree of bias.

The sample sizes in some trials were small. The issue of statistical power in studies evaluating infant formulae has been addressed by a number of scientific organizations. According to ESPGHAN, as a minimum, the study should have a power to detect a difference in weight gain equal to 0.5 SD [15]. The IOM stated that the sample sizes must be large enough to ensure sufficient statistical power in follow-up studies, particularly if these studies are carried out years after the child has ceased infant formula consumption. The IOM recommends a sample size of 52 children per group to detect a moderate-effect size difference and a sample size of 140 children per group to detect an intermediate-effect size difference (i.e., between small and moderate) (with 80% power). The IOM also stated that *‘unless there are compelling reasons to do otherwise, the committee recommends having sufficient power to detect differences between groups of 0.20 SD or less when estimating sample-size needs in follow-up studies.’* The IOM suggests that ‘*even effect sizes of this magnitude can have important clinical implications’*[1]. Considering the fact that the included studies were often too small with insufficient power to identify relevant effects on growth, and the follow-up periods in the trials were short, the findings needs to be interpreted with caution.

Finally, to assess growth, the duration of the study should be at least 3 months, and the IOM has recommended a 120-day growth study to assess the ability of an infant formula to sustain normal growth [1]. In the included trials, the duration of the intervention in some of the studies was much shorter.

### Comparison with other studies

The findings of this systematic review are in line with the previous report by ESPGHAN [2]. Also, a more recent systematic review (search date: 2010) found that probiotic supplementation did not have any significant effect on growth (weight gain, length gain, or head circumference) in boys or girls [16]. None of the included studies reported weight loss. As with this review, the authors noted that the studies had small sample sizes and short follow-up periods. In contrast to this review, all probiotics were evaluated together as a class of agents, with no analyses based on only one type of probiotic microorganism.

Beyond early infancy (starting >4 months of age), evidence from 4 RCTs [17-20], previously reviewed by ESPGHAN, suggests that *B lactis* supplementation (in combination with *Str thermophilus* with or without *L helveticus)* of formula is also associated with adequate growth. No new studies that analyzed the effect of *B lactis* supplementation of formula beyond early infancy have been published since then. However, as previously recommended by ESPGHAN, caution is needed when interpreting these results for several reasons. First, due to the methodological limitations of the study and a high risk of bias. Second, caution is needed when interpreting these results due to the wide age range (from 6 to 36 months). Considering the differences in growth velocity and regulation of growth in young infants and toddlers, no firm conclusions can be made [2]. No data on the effects of LGG supplementation were identified.

Finally, recent evidence has suggested that compared with higher protein content in infant formulae, lower protein content is associated with a lower weight in infants up to 2 years of age [21]. The protein content in the included RCTs differed, and it ranged from 1.8 g of protein/100 kcal to 2.2 g of protein/100 kcal. One may speculate that these differences may have had an impact on growth. However, as the data are limited, and no direct comparisons of a high- *vs.* low-protein formula were made, no firm conclusions can be made based on this review. Considering the potential anti-obesity effect of a low-protein infant formula, such studies are warranted.

## Conclusions

The effect on growth is an important part of the safety evaluation of any product used in infants [22]. Supplementation of infant formulae with *B lactis* results in growth similar to what is found in infants fed unsupplemented formulae. Limited data do not allow one to reach a conclusion regarding the effect of LGG supplementation on infant growth.

## Abbreviations

B lactis: *Bifidobacterium lactis* Bb12; BMI: Body mass index; CI: Confidence interval; DXA: Dual-energy X-ray absorptiometry; ESPGHAN: European Society for Paediatric Gastroenterology, Hepatology and Nutrition; IOM: Institute of Medicine; LGG: *Lactobacillus rhamnosus* GG; NNI: Nestle Nutrition Institute; MD: Mean difference; RCT: Randomized controlled trial.

## Competing interests

HS has participated as a clinical investigator, and/or advisory board member, and/or consultant, and/or speaker for Arla, Biogaia, Biocodex, Danone, Dicofarm, Nestle, Nestle Nutrition Institute, Nutricia, Mead Johnson, and Sequoia. AC has participated as a clinical investigator for Danone.

## Authors’ contributions

HS initially conceptualized this study. Both authors were responsible for data collection, data analysis, data interpretation, and preparation of the report. HS assumed the main responsibility for the writing of this manuscript and is guarantor. Both authors read and approved the final manuscript.

## Pre-publication history

The pre-publication history for this paper can be accessed here:

http://www.biomedcentral.com/1471-2431/13/185/prepub

## Supplementary Material

Additional file 1: Table S1 Pubmed search. Click here for file

Additional file 2: Figure S1 Flow diagram for study selection process. Click here for file

Additional file 3: Table S2 Characteristics of excluded trials with reasons for exclusion. Click here for file

Additional file 4: Figure S2 B lactis vs. control. Administration started in infants <4 mo of age. Outcome: percentiles. Click here for file

Additional file 5: Figure S3 LGG vs. control. Outcomes: weight (g), length (mm), head circumference (mm) at entry, at 3 months, and at 6 months of age. Click here for file

Additional file 6: Figure S4 LGG vs. control. Outcome: change in standard deviation score (SDS). Click here for file
