# Supplementary material for: Growth of infants fed formula supplemented with Bifidobacterium lactis Bb12 or Lactobacillus GG: a systematic review of randomized controlled trials
Source: BMC Pediatr. 2013 Nov 12;13:185. doi: 10.1186/1471-2431-13-185 (PMC3831250; doi:10.1186/1471-2431-13-185)
Supplement: Additional file 1: Table S1 — Pubmed search. [file 1471-2431-13-185-S1.doc]

**Additional file 1: Table S1.** Pubmed search

| #11 | [Add](http://www.ncbi.nlm.nih.gov/pubmed/advanced) | Search **(((#2) AND #3) AND #5) NOT #4** Filters: **Clinical Trial; Review; Humans** | [203](http://www.ncbi.nlm.nih.gov/pubmed/?cmd=HistorySearch&querykey=11) | 06:58:47 |
| --- | --- | --- | --- | --- |
| [#10](http://www.ncbi.nlm.nih.gov/pubmed/advanced) | [Add](http://www.ncbi.nlm.nih.gov/pubmed/advanced) | Search **((#2) AND #3) AND #5** Filters: **Clinical Trial; Review; Humans** | [363](http://www.ncbi.nlm.nih.gov/pubmed/?cmd=HistorySearch&querykey=10) | 06:57:52 |
| [#9](http://www.ncbi.nlm.nih.gov/pubmed/advanced) | [Add](http://www.ncbi.nlm.nih.gov/pubmed/advanced) | Search **(((#2) AND #3) AND #4) AND #5** Filters: **Clinical Trial; Review; Humans** | [160](http://www.ncbi.nlm.nih.gov/pubmed/?cmd=HistorySearch&querykey=9) | 06:56:56 |
| [#8](http://www.ncbi.nlm.nih.gov/pubmed/advanced) | [Add](http://www.ncbi.nlm.nih.gov/pubmed/advanced) | Search **(((#2) AND #3) AND #4) AND #5** Filters: **Clinical Trial; Review** | [161](http://www.ncbi.nlm.nih.gov/pubmed/?cmd=HistorySearch&querykey=8) | 06:56:47 |
| [#7](http://www.ncbi.nlm.nih.gov/pubmed/advanced) | [Add](http://www.ncbi.nlm.nih.gov/pubmed/advanced) | Search **(((#2) AND #3) AND #4) AND #5** Filters: **Clinical Trial** | [107](http://www.ncbi.nlm.nih.gov/pubmed/?cmd=HistorySearch&querykey=7) | 06:56:28 |
| [#6](http://www.ncbi.nlm.nih.gov/pubmed/advanced) | [Add](http://www.ncbi.nlm.nih.gov/pubmed/advanced) | Search **(((#2) AND #3) AND #4) AND #5** | [410](http://www.ncbi.nlm.nih.gov/pubmed/?cmd=HistorySearch&querykey=6) | 06:56:21 |
| [#5](http://www.ncbi.nlm.nih.gov/pubmed/advanced) | [Add](http://www.ncbi.nlm.nih.gov/pubmed/advanced) | Search **(growth or anthropometry or weight or lenght or head circumference or development or physical development)** | [3672743](http://www.ncbi.nlm.nih.gov/pubmed/?cmd=HistorySearch&querykey=5) | 06:56:20 |
| [#4](http://www.ncbi.nlm.nih.gov/pubmed/advanced) | [Add](http://www.ncbi.nlm.nih.gov/pubmed/advanced) | Search **(formula or formulae or milk)** | [148013](http://www.ncbi.nlm.nih.gov/pubmed/?cmd=HistorySearch&querykey=4) | 06:56:19 |
| [#3](http://www.ncbi.nlm.nih.gov/pubmed/advanced) | [Add](http://www.ncbi.nlm.nih.gov/pubmed/advanced) | Search **newborn or infant or infant* or infants or child or children or child*** | [2429957](http://www.ncbi.nlm.nih.gov/pubmed/?cmd=HistorySearch&querykey=3) | 06:56:19 |
| [#2](http://www.ncbi.nlm.nih.gov/pubmed/advanced) | [Add](http://www.ncbi.nlm.nih.gov/pubmed/advanced) | Search **Bifidobacterium or Bifidobacterium bifidum or B bifidum or B.bifidum or B lactis or B. lactis or B lactis Bb12 or B. lactis Bb12 or Bifidobacterium animalis or B. animalis or Bifidobacterium animalis ssp lactis or CNCMI-3446 or Lactobacillus or Lactobacillus rhamnosus or Lactobacillus rhamnosus GG or L. rhamnosus GG or LGG** | [26033](http://www.ncbi.nlm.nih.gov/pubmed/?cmd=HistorySearch&querykey=2) | 06:56:18 |
