# Supplementary material for: Growth of infants fed formula supplemented with Bifidobacterium lactis Bb12 or Lactobacillus GG: a systematic review of randomized controlled trials
Source: BMC Pediatr. 2013 Nov 12;13:185. doi: 10.1186/1471-2431-13-185 (PMC3831250; doi:10.1186/1471-2431-13-185)
Supplement: Additional file 2: Figure S1 — Flow diagram for study selection process. [file 1471-2431-13-185-S2.docx]

**Additional file 2: Figure S1. Flow diagram for study selection process.**

Records identified at first search
(n = 363)

Titles and abstracts screened
(n = 363)

Records excluded
(n = 338)

Full text papers excluded (n =16)

Reasons for exclusion:

Mode or timing of probiotic administration (n=7); Unclear intervention (n=1); Population: children with chronic conditions (n=2); Outcomes: growth not reported (n=6).

Papers included
(n = 9)

Full text papers assessed for eligibility
(n = 25)
