# Supplementary material for: Growth of infants fed formula supplemented with Bifidobacterium lactis Bb12 or Lactobacillus GG: a systematic review of randomized controlled trials
Source: BMC Pediatr. 2013 Nov 12;13:185. doi: 10.1186/1471-2431-13-185 (PMC3831250; doi:10.1186/1471-2431-13-185)
Supplement: Additional file 3: Table S2 — Characteristics of excluded trials with reasons for exclusion. [file 1471-2431-13-185-S3.docx]

**Additional file 3: Table S2.** Characteristics of excluded trials with reasons for exclusion

| **Study** | **Reason for exclusion** |
| --- | --- |
| Bakker-Zierikzee 2005 [^[[1]](#endnote-1)^] | Outcomes: growth not reported (Bb12). |
| Bakker-Zierikzee 2006 [^[[2]](#endnote-2)^] | Outcomes: growth not reported (Bb12). |
| Brouwer 2006 [^[[3]](#endnote-3)^] | Population: infants with atopic dermatitis (LGG). |
| Chouraqui 2004 [^[[4]](#endnote-4)^] | Intervention: formula supplemented with Bb12 & *Str thermophilus* & *L helveticus;* administration beyond early infancy. |
| Langhendries 1995 [^[[5]](#endnote-5)^] | Intervention: formula supplemented with Bb12 & *Str thermophilus* & *L helveticus.* |
| Nopchinda 2002 [^[[6]](#endnote-6)^] | Intervention: formula supplemented with Bb12 & *Str thermophilus;* administration beyond early infancy. |
| Petschow 2005 [^[[7]](#endnote-7)^] | Growth not reported (LGG). |
| Phuapradit 1999 [^[[8]](#endnote-8)^] | Outcomes: growth not reported (Bb12). |
| Rautava 2006 [^[[9]](#endnote-9)^] | Outcomes: growth not reported (Bb12+LGG). |
| Rautava 2009 [^[[10]](#endnote-10)^] | Outcomes: growth not reported (Bb12+LGG). |
| Rinne 2005 [^[[11]](#endnote-11)^] | Intervention: probiotic (LGG) dissolved in water given in addition to breast milk. |
| Rochat 2007 [^[[12]](#endnote-12)^] | Intervention: no probiotic in study formula (trial included in the review by Steenhout et al.^[[13]](#endnote-13)^ as Bb12-containing formula). The data from this study are now included in the study by Barclay et al.^[[14]](#endnote-14)^ |
| Saavedra 1994 [^[[15]](#endnote-15)^] | Population: chronically ill children (*B bifidum* and *Str thermophilus)* |
| Saavedra 2004 [^[[16]](#endnote-16)^] | Intervention: formula supplemented with Bb12 & *Str thermophilus;* administration beyond early infancy. |
| Taipale 2011 [^[[17]](#endnote-17)^] | Intervention: probiotic (Bb12) released by a special pacifier; mainly in breast-fed infants. |
| Weizman [^[[18]](#endnote-18)^] | Intervention: formula supplemented with Bb12*;* administration beyond early infancy. |

LGG, *Lactobacillus* GG; Bb12 *– B lactis* Bb12

**REFERENCES**

1. Bakker-Zierikzee AM, Alles MS, Knol J, Kok FJ, Tolboom JJ, Bindels JG. Effects of infant formula containing a mixture of galacto- and fructo-oligosaccharides or viable Bifidobacterium animalis on the intestinal microflora during the first 4 months of life. Br J Nutr 2005;94:783-90. [↑](#endnote-ref-1)
2. Bakker-Zierikzee AM, Tol EA, Kroes H, Alles MS, Kok FJ, Bindels JG. Faecal SIgA secretion in infants fed on pre- or probiotic infant formula. Pediatr Allergy Immunol 2006;17:134-40. [↑](#endnote-ref-2)
3. Brouwer ML, Wolt-Plompen SA, Dubois AE, van der Heide S, Jansen DF, Hoijer MA, Kauffman HF, Duiverman EJ. No effects of probiotics on atopic dermatitis in

   infancy: a randomized placebo-controlled trial. Clin Exp Allergy 2006;36:899-906. [↑](#endnote-ref-3)
4. Chouraqui JP, Van Egroo LD, Fichot MC. Acidified milk formula supplemented with bifidobacterium lactis: impact on infant diarrhea in residential care settings. J Pediatr Gastroenterol Nutr 2004;38:88–92**.** [↑](#endnote-ref-4)
5. Langhendries JP, Detry J, Van Hees J, et al . Effect of a fermented infant formula containing viable bifidobacteria on the fecal flora composition and pH of healthy full-term infants. J Pediatr Gastroenterol Nutr 1995;21:177-81. [↑](#endnote-ref-5)
6. Nopchinda S, Varavithya W, Phuapradit P, et al. Effect of *Bifidobacterium* Bb12 with or without *Streptococcus* thermophilus supplemented formula on nutritional status. J Med Assoc Thai 2002;85(Suppl 4):S1225–31**.** [↑](#endnote-ref-6)
7. Petschow BW, Figueroa R, Harris CL, Beck LB, Ziegler E, Goldin B. Effects of feeding an infant formula containing *Lactobacillus* GG on the colonization of the intestine: a dose-response study in healthy infants. J Clin Gastroenterol 2005;39:786-90. [↑](#endnote-ref-7)
8. Phuapradit P, Varavithya W, Vathanophas K, Sangchai R, Podhipak A, Suthutvoravut U, Nopchinda S, Chantraruksa V, Haschke F. Reduction of rotavirus infection in children receiving bifidobacteria-supplemented formula. J Med Assoc Thai 1999;82 Suppl 1:S43-8. [↑](#endnote-ref-8)
9. Rautava S, Arvilommi H, Isolauri E. Specific probiotics in enhancing maturation of IgA responses in formula-fed infants. Pediatr Res 2006;60:221-4. [↑](#endnote-ref-9)
10. Rautava S, Salminen S, Isolauri E. Specific probiotics in reducing the risk of acute infections in infancy--a randomised, double-blind, placebo-controlled study. Br J Nutr 2009;101:1722-6. [↑](#endnote-ref-10)
11. Rinne MM, Gueimonde M, Kalliomäki M, Hoppu U, Salminen SJ, Isolauri E. Similar

    bifidogenic effects of prebiotic-supplemented partially hydrolyzed infant formula and breastfeeding on infant gut microbiota. FEMS Immunol Med Microbiol 2005;43:59-65. [↑](#endnote-ref-11)
12. Rochat F, Cherbut C, Barclay D. A whey-predominant formula induces fecal microbiota similar to that found in breast-fed infants. Nutr Res 2007;27:735–40. [↑](#endnote-ref-12)
13. Steenhout PG, Rochat F, Hager C. The effect of *Bifidobacterium lactis* on the growth of infants: a pooled analysis of randomized controlled studies. Ann Nutr Metab 2009;55:334-40. [↑](#endnote-ref-13)
14. Barclay D, Puccio G, Fazzolari-Nesci A, et al. Growth and tolerance of a whey-based starter infant formula with enhanced protein efficiency and containing pro-, pre or synbiotics. A randomized controlled trial in term infants. J Pediatr Gastroenterol Nutr 2003;37:388. Abstract. [↑](#endnote-ref-14)
15. Saavedra J, Bauman NA, Oung I, et al. Feeding of *Bifidobacterium bifidum* and *Streptococcus thermophilus* to infants in hospital for prevention of diarrhea and shedding of rotavirus. Lancet 1994;344:1046–9**.** [↑](#endnote-ref-15)
16. Saavedra JM, Abi-Hanna A, Moore N, et al. Long-term consumption of infant formulas containing live probiotic bacteria: tolerance and safety. Am J Clin Nutr 2004;79:261–7**.** [↑](#endnote-ref-16)
17. Taipale T, Pienihäkkinen K, Isolauri E, Larsen C, Brockmann E, Alanen P, Jokela J, Söderling E. *Bifidobacterium animalis* subsp. *lactis* BB-12 in reducing the risk of infections in infancy. Br J Nutr 2011;105:409-16. [↑](#endnote-ref-17)
18. Weizman Z, Asli G, Alsheikh A. Effect of a probiotic infant formula on infections in child care centers: comparison of two probiotic agents. Pediatrics 2005;115:5–9**.** [↑](#endnote-ref-18)
