# Supplementary material for: Growth of infants fed formula supplemented with Bifidobacterium lactis Bb12 or Lactobacillus GG: a systematic review of randomized controlled trials
Source: BMC Pediatr. 2013 Nov 12;13:185. doi: 10.1186/1471-2431-13-185 (PMC3831250; doi:10.1186/1471-2431-13-185)

**Additional file 4: Figure S2.** *B lactis* vs. control. Administration started in infants <4 mo of age. Outcome: percentiles.


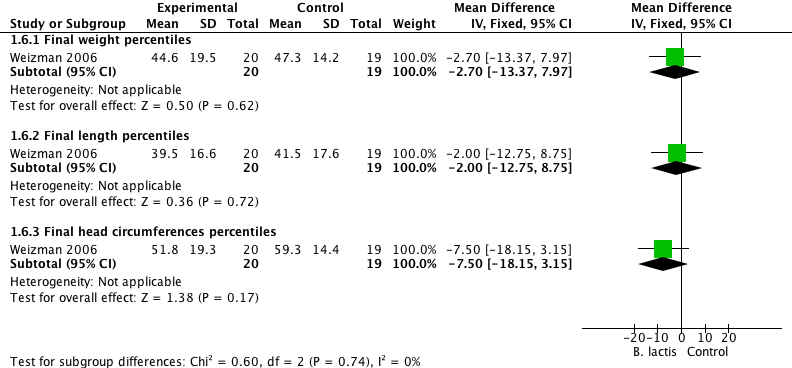

Supplement: Additional file 4: Figure S2 — B lactis vs. control. Administration started in infants <4 mo of age. Outcome: percentiles. [file 1471-2431-13-185-S4.doc]
