# Supplementary material for: Growth of infants fed formula supplemented with Bifidobacterium lactis Bb12 or Lactobacillus GG: a systematic review of randomized controlled trials
Source: BMC Pediatr. 2013 Nov 12;13:185. doi: 10.1186/1471-2431-13-185 (PMC3831250; doi:10.1186/1471-2431-13-185)
Supplement: Additional file 5: Figure S3 — LGG vs. control. Outcomes: weight (g), length (mm), head circumference (mm) at entry, at 3 months, and at 6 months of age. [file 1471-2431-13-185-S5.doc]

**Additional file 5: Figure S3.** LGG vs. control. Outcomes: weight (g), length (mm), head circumference (mm) at entry, at 3 months, and at 6 months of age

**
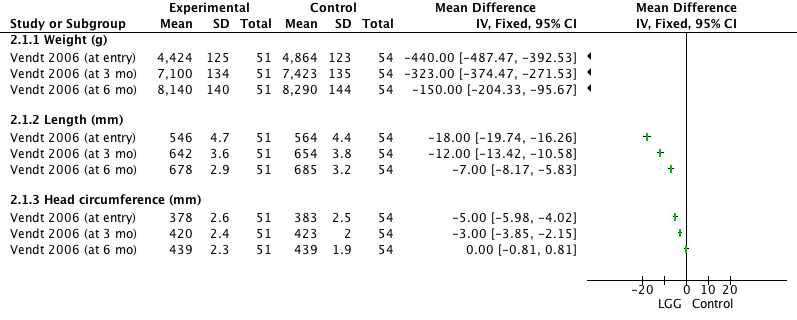
**
