# Supplementary figures and images for: Growth of infants fed formula supplemented with Bifidobacterium lactis Bb12 or Lactobacillus GG: a systematic review of randomized controlled trials
Source: BMC Pediatr. 2013 Nov 12;13:185. doi: 10.1186/1471-2431-13-185 (PMC3831250; doi:10.1186/1471-2431-13-185)

**Additional file 6: Figure S4.** LGG vs. control. Outcome: change in standard deviation score (SDS)

**
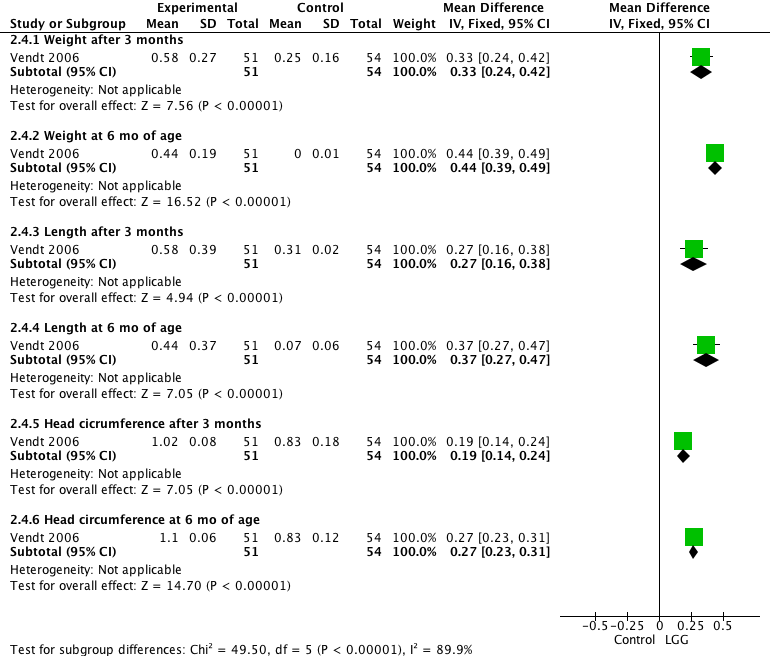
**

Supplement: Additional file 6: Figure S4 — LGG vs. control. Outcome: change in standard deviation score (SDS). [file 1471-2431-13-185-S6.doc]
